# Description and comparison of postoperative functioning of patients with hip fracture 2018 and 2008 at the Örebro University Hospital - a comparative cross-sectional study

**DOI:** 10.1186/s12877-022-03553-y

**Published:** 2022-11-08

**Authors:** Amanda Hammer, Katarina Ljungberg, Tony Bohman, Åsa G Andersson

**Affiliations:** 1 Department of Geriatrics, Örebro University Hospital, Örebro, Sweden; 2 Skövde rehabmottagning, Närhälsan, Skövde, Sweden; 3 Dalarna University, Falun, Karolinska Institutet, Stockholm, Sweden; 4 Department of Geriatrics, Faculty of Medicine and Health, Örebro University, Örebro, Sweden

**Keywords:** Walking ability, Physical activity, Handgrip strength, Multimorbidity, Regression analysis

## Abstract

**Background:**

Hip fractures are a global problem, and it will probably increase. Hip fractures impair health aspects which creates demands on postoperative care. This study describes and compares patients with hip fracture in 2008 and in 2018. An increased knowledge of this group could be a basis how to optimize aftercare and dimension rehabilitation.

**Methods:**

Using a comparative cross-sectional study to describe and compare patients with hip fracture from 2018 and 2008 at Örebro University Hospital regarding age, sex, multimorbidity, fracture type, surgical materials, surgery within 24 hours, length of stay, postoperative walking ability, physical activity level and hand grip strength. Data was collected from 76 patients with hip fracture from 2018 and 78 patients from 2008. Outcome measures considering functioning were walking ability (Functional Ambulation Categories), physical activity level (Classification system of physical activity) and hand grip strength (Jamar hand dynamometer). Statistical analyses used were hypothesis tests and regressions analysis.

**Results:**

No differences in age, sex, fracture type, proportion of surgery within 24 hours or length of stay between the cohorts. The cohort 2018 had more multimorbidity in number of diagnoses and ASA-classification preoperatively. In 2018 70% of the participants were dependent in walking ability (physical human support) compared to 43% 2008 (*p* = 0.007). Proportion of physically inactive was 9% in 2018 compared to 21% 2008 (*p* = 0.047). Hand grip strength was 5.1 kg better in 2018 (*p* = 0.011). Adjusted for age, sex, ASA-classification (American Society of Anaesthesiologists Classification System), surgical materials and number of days between surgery and testing the cohort of 2018 had a lower odds to have independent walking ability and higher odds to be physical active. Differences in hand grip strength decreased to 4.7 kg. Participants in 2018 suffered significantly more multimorbidity.

**Conclusions:**

Study indicated differences in patients’ postoperative functioning between 2018 and 2008 with more impaired walking ability, more multimorbidity, higher proportion of physically active and better hand grip strength 2018. The results are important for future reasoning regarding care needs of patients with hip fracture.

## Introduction

Hip fractures are a global problem that is feared to increase [1]. The incidence of hip fractures worldwide was estimated at 1.6 million in 1990 and is expected to rise to 6.3 million by 2050 [1]. In Sweden, approximately 18.000 people suffer a hip fracture every year [2]. Most hip fractures are caused by low-energy trauma, usually in connection to indoor fall accidents [2]. The risk of a hip fracture increases with age and reduced functioning [2, 3]. Age-related disability can be caused by sarcopenia, fragility, osteoporosis and disease-related malnutrition [3]. A hip fracture is verified by X-ray examination and classified into different fracture types: femoral neck, pertrochanteric femoral and subtrochanteric femoral [2]. Treatment is in most cases surgery, aiming to stabilize the fracture and facilitate patient mobilisation post-surgery [2]. Fast surgery and early mobilisation aim to enable the patient to return to an equivalent functioning and quality of life as before the fracture [4].

A large proportion of patients with hip fracture never regain the functioning they had before the fracture [2, 5]. Zusman et al. claim that people who have had hip fracture surgery often have very limited physical activity and spend a large part of the day lying or sitting even 1 year after the fracture [6]. Many of those affected by hip fracture are multimorbid, which can affect rehabilitation [4]. Therefore, it is important to assess and measure body function and activity level in these patients [7]. Studies concerning healthy populations, shows that hand grip strength correlates to muscle strength in the lower extremities, and that reduced hand grip strength increases the risk of mortality [8, 9]. Regarding hand grip strength and physical activity after a hip fracture, studies show trends with a deterioration in performance over time for patients with hip fracture [10, 11].

In a study from Lund, Sweden, no effect on the functional level on patients with hip fracture was demonstrated over a 25-year period despite improvements in surgical technique and patient management [12]. Another study from Lund showed a significant increase in dependent walking and worse health status with a higher proportion in the higher ASA- classification (American Society of Anaesthesiologists Classification System) 2017 compared to 1999 [13]. Rikshöfts Annual Report of 2017 shows that 4 months after hip fracture, the walking ability remains relatively unchanged over the last 30 years [5].

When planning this study literature searches did not result in any studies comparing the development over time for newly operated patients with hip fracture in terms of postoperative hand grip strength, walking ability and physical activity level. These three factors can be considered central to the functional condition of patients with hip fracture and therefore relevant to study.

An increased knowledge of the patient group’s demographics, multimorbidity, postoperative functional conditions and care needs could be a basis for how we may need to optimize aftercare in the future and dimension rehabilitation and health needs.

The aim of this study was to describe and compare patients with hip fracture in 2018 and 2008 at the University Hospital Örebro (USÖ) Sweden in terms of age, sex, multimorbidity, fracture type, surgical material, surgery within 24 hours, care time, postoperative walking ability, physical activity level and hand grip strength.

## Materials and methods

### Study design and population

In this comparative cross-sectional study, all patients undergoing surgery at USÖ due to hip fracture diagnosed with ICD-10 codes S72.0, S72.1 or S72.2 during 5 months in 2008 and in 2018 respectively were invited to participate. There were no exclusion criteria. All participants received verbal information. Informed consent was obtained from all subjects and they gave their written consent to participate.

### Measurement methods and outcomes

Age was calculated based on the year of birth. The sex was female or male. Multimorbidity was the number of diagnoses in addition to the hip fracture diagnosis, and the ASA-classification. The ASA-classification gave an idea of the patient’s preoperative health [14]. It is a six-point scale (1–6, where 1 means an otherwise healthy patient and 6 means that the patient is classified brain dead) [14]. The fracture types included were femoral neck, pertrochanteric femoral and subtrochanteric femoral. The surgical materials used have been grouped into prosthesis (total and hemi), osteosynthesis material (all bolt, screws and plates) and flail joint. Surgery within 24 hours, from X-ray to start of surgery, was divided into two groups, yes or no. Length of stay meant the number of days in the orthopedic care unit.

Functional Ambulation Categories (FAC) is an assessment scale for categorizing walking and the need of human physical support when walking (0–5, where 0 means cannot walk or requires help of two or more people and 5 means independent walkers) [15]. Classification System of Physical Activity (CSPA) is a scale used to assess the level of physical activity and household activities [16, 17]. It is a six-point scale (1–6, where 1 means barely physically active and 6 involves hard regular training [16, 17]. A modification of the scale was used where physical and household activity were separated and a 0 level was added (not physically active). Household activities were not analyzed. Hand grip strength was measured with a hand dynamometer (Jamar), where hand grip strength was measured in kilograms (kg) [18]. The best attempt of three on the best hand was evaluated. Number of days was used to record time between surgery and the respective assessment of FAC, CSPA and hand grip strength.

### Data collection methods

Age, sex, multimorbidity, fracture type, surgical material, surgery within 24 hours and length of stay were collected from patient records. Assessment of FAC, CSPA and hand grip strength was carried out by licensed physiotherapists. Both data collections were conducted in clinical everyday life and the measurements were done at the nursing department during the patients hospital stay. The participants were not send to a specific lab or testing centre for the measurements. The postoperative care regarding rehabilitation did not follow any standardized program in either 2008 or 2018, but focus was on avoiding postoperative complications (for example pneumonia, atelectasis, pressure ulcers, contractions etc) and start the rehabilitation by promoting early mobilization and active movement training.

### Statistical analysis methods

Patients not able to perform the assessments were not used in the comparative analyses. Two dropout analyses have been carried out. The first was the included and not included patients in both cohorts and they were analyzed regarding differences in sex and age data. The second one, patients able to perform or not perform hand grip strength measurement, in cohort 2018, were compared regarding age, sex, number of diagnoses and ASA-classification. Independent T-test was used to compare differences regarding age, length of stay, hand grip strength and number of days between surgery and assessment [19]. For comparison of differences regarding sex, fracture type, surgical material, surgery within 24 hours and walking ability, chi-2-test was used [19]. For comparison of differences regarding number of diagnoses, ASA classification and physical activity level, Mann-Whitney u-test was used [19]. The significance level was set to *p* < 0.05 in all tests. Furthermore, regression analyses were used to account for potential confounding effects [19]. The outcome FAC was dichotomized into dependent (FAC scale steps 0–3) and independent (FAC scale steps 4–5) walkers and analysed with logistic regression. The outcome CSPA was dichotomized into not physical active (scale step 0) and physical active (scale steps 1–3) and analysed with logistic regression. The continuous outcome hand grip strength was analysed using linear regression. Five confounders (age, sex, ASA-classification, surgical material and number of days between surgery and measurement) were included separately one by one in the respective regression and if the affected outcome was 10% or more it was included in the final multiple regression [20, 21]. Finally, a multiple regression analysis was performed with all the confounders that affect the outcome [20]. A 95% confidence interval was chosen and when calculating the odds ratio, a 95% confidence interval (CI) is statistically significant if it does not contain 1.0 [22]. The IBM SPSS Statistics software for Windows, version 26, was used for all statistical analyses.

### Patient and public involvement

No patient involved.

## Results

### Background data study participants

During the inclusion period in 2008, a total of 108 people underwent surgery, of which 78 people agreed to participate in the study. During the inclusion period in 2018, a total of 97 people had surgery, of which 76 people agreed to participate. Of the 30 and 21 who did not participate, the major reason was impaired ability to give consent due to cognitive state.

There was no difference in sex between included and not included in 2008 (*p* = 0.961) or 2018 (*p* = 0.696). The average age of those included in 2008 was 81 years and for non-included 84 years (*p* = 0.256). In 2018, the average age was 80 and those not included were 87 years (*p* = 0.007).

Table 1 shows background data for participants in 2008 and 2018. There was no statistically significant difference between the cohorts in terms of age, sex, fracture type, type of surgical material, surgery within 24 hours or length of stay in the orthopedic care unit. Regarding the number of diagnoses, participants in 2018 had one additional diagnosis on average, (*p* = 0.052). Regarding ASA-classification, there was a significant difference, (*p* < 0.001), where participants in 2018 were classified with poorer health conditions.

**Table 1:** Baseline characteristics of cohort 2008 and cohort 2018

	Cohort 2008 *n* = 78	Cohort 2018 *n* = 76	*P-value*
**Age**, mean (SD), years	81 (11)	80 (12)	0.677
**Gender**, *n* (%)			
Female	49 (63)	47 (62)	0.900
Men	29 (37)	29 (38)	
**Number of diagnoses**, median (IQR)	3 (4)	4 (4)	0.052
**ASA-classification**, *n* (%)			
ASA-class 1	8 (10)	5 (7)	
ASA-class 2	40 (51)	25 (33)	< 0.001^a^
ASA-class 3	26 (34)	34 (44)	
ASA-class 4	1 (1)	12 (16)	
Unspecified	3 (4)	0	
**Fracture type**			
S72.0 (femoral neck fracture)	41 (52)	37 (49)	
S72.1 (subtrochanteric femoral fracture)	31 (40)	31 (41)	0.793
S72.2 pertrochanteric femoral fracture)	6 (8)	8 (10)	
**Type of surgical material**, *n* (%)			
Prosthesis	13 (17)	20 (26)	
Osteosynthesis material	65 (83)	55 (72)	0.193
Flail joint	0	1 (2)	
**Surgery within 24 hours**, *n* (%)			
Yes	37 (48)	38 (50)	
No	40 (51)	37 (49)	0.949
Not specified	1 (1)	1 (1)	
**Length of stay**, mean (SD), days	10 (5)	9 (4)	0.308

*Abbreviations*: *SD* standard deviation, *IQR* interquartile range, *ASA-classification* American Society of Anaesthesiologists Classification System

^a^ significant

### Postoperative walking ability according to FAC

Postoperative walking ability according to FAC were analyzed in cohort 2008 for 75 participants with loss of three (one early death, one discontinued participation and one unspecified) and in cohort 2018 for 69 participants with loss of seven (one with reduced general condition, time constraints for a staff of four and two unspecified).

**Fig. 1:**
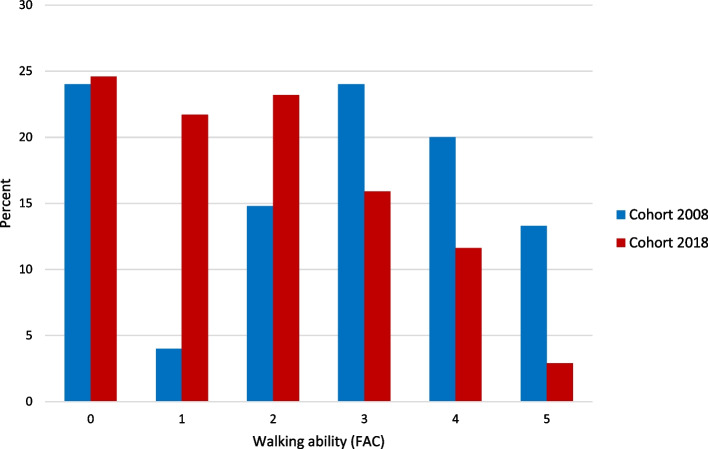
Postoperative walking ability measured with Functional Ambulation Categories (FAC) reported in the proportion of participants (%) for each scale step. The scale reaches from 0 to 5, where 0 means cannot walk or requires help of two or more people and 5 means independent walkers

Figure 1 shows postoperative walking ability according to FAC for participants in 2008 and 2018. In 2008, 43% were dependent on human physical support when walking (FAC 0 to 2) compared to 70% in 2018. In 2008 the proportion independent of human physical support when walking (FAC 3 to 5) was 57%, of which 24% needed supervision or verbal instructions. In 2018 the proportion independent of human physical support when walking (FAC 3 to 5) was 30%, of which 16% needed supervision or verbal instructions. The median for participants in 2008 was at scale step 3 (IQR 3) compared to scale step 2 (IQR 3) in 2018. When comparing the cohorts, there was a statistically significant difference in walking ability according to FAC, (*p* = 0.007), where the 2018 participants had a more impaired walking ability.

In 2008 the number of days between surgery and assessment of walking ability according to FAC was on average 6 days (SD 2) compared to 5 days (SD 2) in 2018. The difference of 1 day was estimated to be statistically significant (*p* < 0.001).

### Classification system of physical activity postoperative

Postoperative physical activity levels according to CSPA were analyzed in cohort 2008 for 73 participants with a loss of five (two with reduced general condition, one discontinued participation, one early death and one unspecified) and in cohort 2018 for 69 participants with a loss of seven (one with reduced general condition, time constraints for a staff of two and four unspecified).

**Fig. 2:**
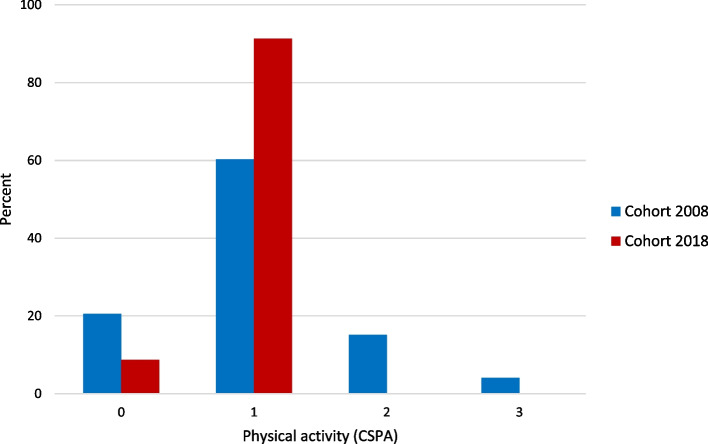
Postoperative physical activity measured with Classification System of Physical Activity (CSPA) and reported in the proportion of participants (%) for each scale step. The scale reaches from 0 to 6 where 0 means no physical activity and 6 hard regular training

Figure 2 shows the percentage of postoperative physical activity for participants in 2008 and 2018. In terms of percentage, twice as many participants in 2008 were completely physically inactive compared to 2018. In 2008, a fifth of participants were more physically active, scale steps 2 and 3 (most sedentary and lighter physical exertion). In 2018, all participants were assessed as scale steps 0 and 1, (not physically active at all or barely physically active). In both cohorts, the median scale step was 1 (IQR 0). When comparing the cohorts, there was a statistically significant difference, (*p* = 0.047), where in 2018 there were more participants who were physically active.

In 2008 the number of days between surgery and physical activity level assessment was on average 6 days (SD 2) compared to 5 days (SD 2) in 2018. The difference of 1 day was estimated to be statistically significant, *p* < 0.001.

### Postoperative hand grip strength

Postoperative hand grip strength was analyzed in cohort in 2008 for 69 participants with a loss of nine (three with reduced general condition, one discontinued participation, one early death and four unspecified) and in cohort 2018 for 57 participants with a loss of 19 (six with reduced general condition, one with impaired cognition, one who declined, at one point the dynamometer was on loan, one patient had cast and peripheral venous catheter, lack of time with staff on six occasions and three unspecified).

In 2018, those who did not participate in the measurement were on average 3 years younger (*p* = 0.258). Among the dropouts, 79% were women, while the corresponding figure for those who performed measured grip strength was 56%, (*p* = 0.076). The median of the drop-out group had one additional diagnosis (*p* = 0.340) and 10% more belonged to ASA-classifications 3 and 4 (*p* = 0.419).

**Fig. 3:**
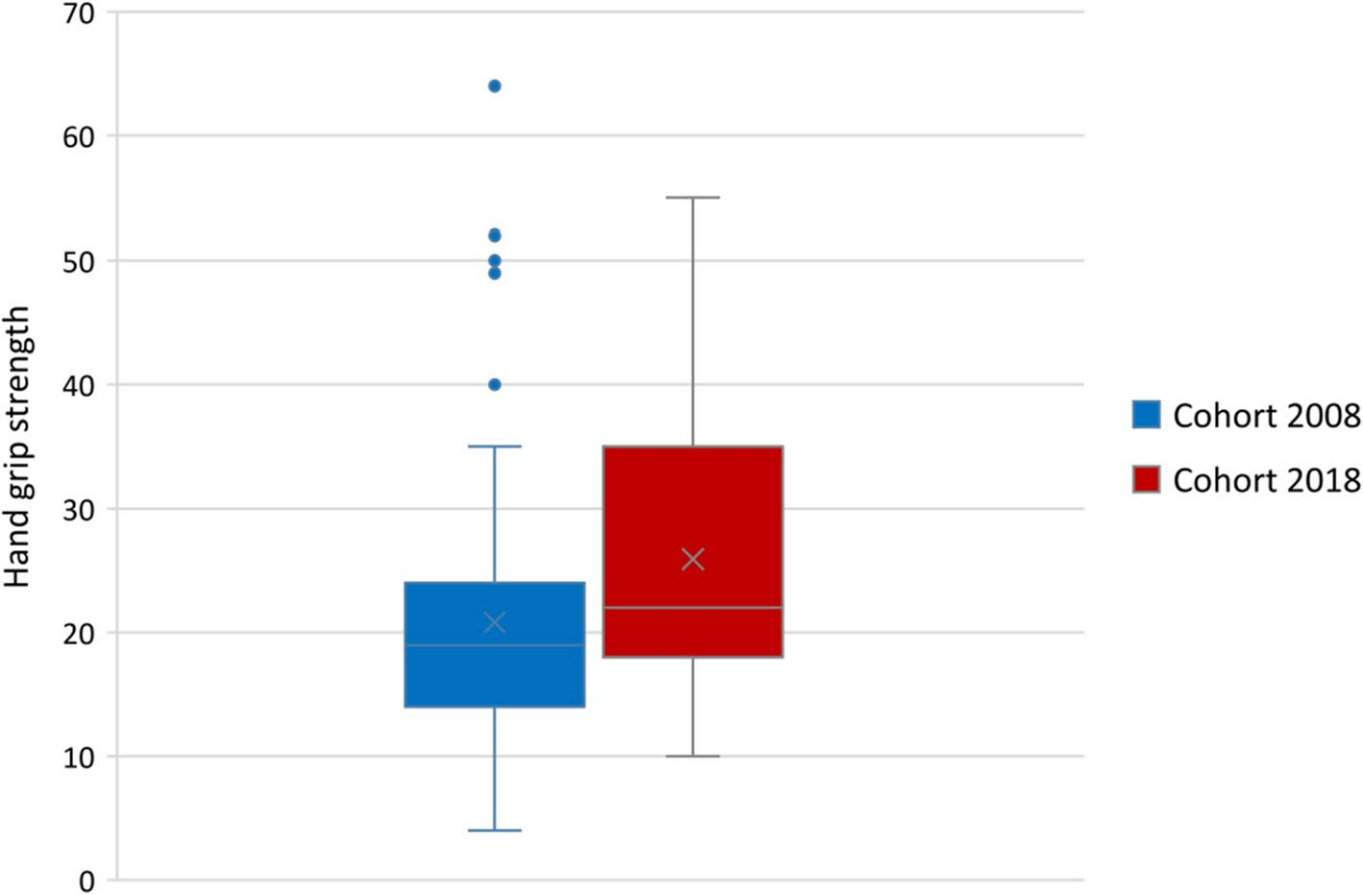
Outcomes of postoperative hand grip strength in kg for cohort 2008 and cohort 2018 respectively

Figure 3 shows postoperative hand grip strength in kg for participants in 2008 and 2018. In the cohort 2008, the hand grip strength was on average 20.8 kg (SD 11) compared to an average of 25.9 kg (SD 11) in cohort 2018. When compared, participants were on average 5.1 kg stronger 2018 than in 2008, giving a statistically significant difference (*p* = 0.011).

In 2008 the number of days between surgery and hand grip strength testing was on average 6 days (SD 2) compared to 6 days (SD 4) in 2018, (*p* = 0.153).

### Regression analyses

Table 2 presents results from multiple regression analyses of FAC, CSPA and hand grip strength. Adjusted for confounders, the cohort 2018 had five times lower odds of being independent walkers compared to cohort 2008. The cohort 2018 had 3.6 times higher odds of being physically active compared to the cohort 2008. When comparing hand grip strength between cohorts, participants in 2018 were on average 4.7 kg stronger than in 2008.

**Table 2:** Multiple regression analyses unadjusted and adjusted for confounders that affected the outcome 10% or more

	n=	Unadjusted	Adjusted
**FAC**^a^			
Cohort 2008	75	1,0	1,0
Dependant	32		
Independant	43		
Cohort 2018	69	0.3 (0.2–0.6)	0.2 (0.1–0.4)^c^
Dependant	48		
Independant	21		
**CSPA**^a^			
Cohort 2008	73	1.0	1.0
Not physical active	15		
Physical active	58		
Cohort 2018	69	2.7 (1.0–7.4)	3.6 (1.1–11.9)^d^
Not physical active	6		
Physical active	63		
**Hand grip strength**^b^			
Cohort 2008		0.0	0.0
Cohort 2018		5.1 (1.2–9.0)	4.7 (1.7–7.7)^e^

*Abbreviations*: *FAC* Functional Ambulation Categories, *CSPA* Classification System of Physical Activity, *ASA-classification* American Society of Anaesthesiologists Classification System

^a^ Multiple logistic regression, reported in odds ratio and confidence interval, (CI)

^b^ Multiple linear regression, reported in kg and confidence interval (CI)

^c^ Adjusted for age, surgical material, ASA-classification and number of days between surgery and assessment

^d^ Adjusted for ASA-classification, surgical material and number of days between surgery and assessment

^e^ Adjusted for gender, ASA-classification and number of days between surgery and test

## Discussion

In this study we found no differences in age, sex, fracture type, proportion of surgery within 24 hours or length of stay between the cohorts. The cohort 2018 had more multimorbidity in number of diagnoses and ASA-classification preoperatively. The cohort 2018 had more impaired walking ability, higher proportion of physically active and better handgrip strength postoperatively. Adjusted for confounders, the cohort 2018 had lower odds of being independent walkers, a higher odds of being physically active and were 4.7 kg stronger in hand grip strength.

There were no exclusion criteria, which we believe is a strength as everyone who wanted and could participate in the study was included. Another strength is that the tests were carried out in the clinical everyday life since this was our opportunity to include the complete patient group, which would probably not be possible after discharge. When analyzing those not included, the mean age was statistically significantly higher in 2018. If they had been possible to include, the results might have been worse, as increased age may lead to a worsening of the functional condition and a risk of multimorbidity [23]. In this study, we chose to count the number of diagnoses for each cohort. We believe that it is of greater value to know the number of diagnoses than whether the patient is classified as multimorbid or not. No further research was made which diagnoses the patients had, but this would have been of interest as different diagnoses may have different impact on the recovery of functioning [24]. We are aware that the ASA-classification applies to the patient’s preoperative state of health, but nevertheless believe that it provides important information to postoperative care. Regarding length of stay, only the time of care in the orthopedic unit is referred to. In the majority of cases, the patient’s care time has probably continued in another care unit, municipal care or in primary care. The assessment instrument FAC is not tested for validity or reliability for patients with hip fracture but only for neurological conditions [25]. The assessment scale only evaluates the demand of human physical support the patient needs, it does not consider, for example, which walking aid is used. FAC is perceived to focus on the individual’s balance difficulties when walking, while newly operated patients with hip fracture are usually more limited by pain and fear of movement [4, 5]. The CSPA has undergone some modifications in this study which means that the validity and reliability tests available in previous studies cannot be fully transferred. However, the modifications are considered relevant but should be validated and reliability tested in future studies. Since the assessment is carried out a few days postoperatively, the scale can be roughly divided and not sensitive enough to provide a fair spread. Assessment instruments with greater focus on locomotion might have been more appropriate to use. For example, the New Mobility Score or Cumulated Ambulatory Score, which are validity and reliability tested in patients with hip fracture and recommended by Fitzgerald et al. [26]. Household activities were excluded in this study, as there was no possibility for study participants to perform such activities in a care unit so shortly after surgery. Jamar hand dynamometer is not validated or reliability tested for patients with hip fracture but in elderly ill and also in healthy individuals, which means that transferability is considered good [18]. This study was based on the best effort of the best hand, which represents the patient’s maximum hand grip strength. Similar approaches have been used in other studies, while others suggest that an average of three trials provides better reliability [9, 18]. Measuring grip strength, which according to previous studies correlates well with lower extremity strength, we believe is the most appropriate approach to measuring muscle strength so short in time postoperatively [8]. Regarding the regression analysis, we selected five confounders that we believed could affect the outcome, but there is a risk that other confounders, which could have had an impact, were not controlled for. In order to perform a logistic regression analysis for CSPA, we needed to allocate the scale steps to a binary outcome. Due to the distribution, we needed to have scale step 0 (not physically active) separately in one group and scale steps 1 to 3 (barely physically active to little/moderately physically active) in the second group. We are aware that there was a large variation in the group that includes three scale steps, which challenge that the result was not optimally adjusted. The logistic regression considering FAC were also allocated into a binary outcome. We thought that this grouping was reasonable partly from the enormous difference whether the patient is dependent or independent in their walking ability. This from both the individual patient’s perspective but also the financial consequences that results from if the patient need or do not need help of staff. But we understand that detail information have been lost when dichotomized. The proportion of drop-out of hand grip strength was 12 and 25%, respectively, which may cause the result not optimally reliable [27].

In this study the average age when a hip fracture incurred was 80 years and 81 years respectively, which is consistent with other studies and strengthens that the elderly population are the ones to suffer [5, 12]. Regarding sex distribution in the two cohorts the difference was minimal, in 2018 62% were women compared to 63% in 2008. One explanation that more females are affected may be that more females than men suffer from osteoporosis [28]. There was a higher rate of multimorbidity in 2018 than in 2008 in terms of numbers of diagnoses and ASA-classification, which means that aftercare may need to be more prepared for the more impaired state of health of the population and the recovery may therefore take longer [4]. In 2018, 60% of participants were classified in ASA-class 3 and 4 compared to 35% in 2008, a change of 25%. This is consistent with results from a Swedish study in 2019, in which 55% of patients with hip fracture were classified as ASA 3 and 4 [13]. Femoral neck fracture remains the most common fracture in both cohorts (approximately 50%), pertrochanteric femoral the second most prevalent (approximately 40%) and subtrochanteric femoral are the least frequent (about 10%). This is well in line with most regions of Sweden [5]. The average length of stay from arrival in the emergency room to discharge from the orthopedic unit has decreased by 24 hours during this 10-year period. This can be interpreted as a sign of a well-functioning postoperative care. The length of stay of both cohorts are the same length as in other hospitals in Sweden [4, 5]. Postoperative walking ability has deteriorated over this 10-year period. The results are consistent with another study showing that there is an increasing proportion of dependent walkers [13]. The difference in number of days between surgery and assessment of walking ability was statistically significant and may affect the result, as the participants in 2018 were tested earlier postoperatively and were therefore expected to have a more impaired walking ability. This was controlled in the regression analysis. A more impaired walking ability may also be explained by a more multimorbid patient group, which already may have had a more impaired functional condition and thus having more difficulties to recover postoperatively. This study has not analyzed the preoperative walking ability, which could have demonstrated a more impaired walking ability already preoperatively and allowed for an analysis of the change. The proportion of physically active people was higher in 2018. However, the results showed that the majority of participants in both cohorts had a low level of physical activity. A reasonable result reinforced by Davenport et al. when the assessment was carried out shortly after surgery [29]. The difference in number of days between surgery and physical activity assessment was statistically significant. Had the test been carried out with the same length of time in between both cohorts, perhaps even greater difference would have been demonstrated between the cohorts. When controlling for confounders higher odds was shown for cohort 2018 to be more physically active, despite the fact that in 2008 some participants were assessed to have higher physical activity levels, scale steps 2 and 3. Preoperative activity level is not analyzed, which could have been higher in 2018. The cohort 2018 had better hand grip strength, a difference that was statistically significant. The explanation for the difference is probably multifactorial. For example, more accessible information about diet and exercise, more specifically targeted training for the elderly and improved care of many diseases and diagnoses may be possible explanations. According to other studies, there is a relationship between hand grip strength and lower extremity strength and ADL function, which would mean that in 2018 the participants would also be stronger in the lower extremity and have a better ADL function [8, 9]. However, our results showed that the hand grip strength was better in 2018 while walking ability was more impaired. This may be explained by the fact that it is mainly pain that limits walking ability early postoperatively and not strength [4, 5, 26]. There were no specific rehabilitation program for hip fracture patients in either 2008 or 2018 so the primary goal of early mobilization to avoid postoperative complications, start the rehabilitation and active movement training were the same for both cohorts. The absence of a standardized postoperative rehabilitation program may have allowed patients to have received different postoperative care, which may have affected the result. Since the length of stay was relatively short, we do not believe that the variation in the rehabilitation the patient received was very large as it still needs to be individualized according to the patient’s ability.

The results are considered to have relatively good generalizability to the entire hip fracture population, as there were no exclusion criteria and the cohorts were relatively large. However, we only have data from one hospital which could be considered a possible selection bias. This study shows that more people are dependent on human physical support in 2018, which is important information when reasoning about the future of postoperative care. For example, there may be an increased need for staff in order for more patients to have the opportunity for optimal mobilisation and walking training in inpatient care, primary or municipal care. The continued very low level of physical activity in the population should also be considered in clinical everyday life as early mobilisation postoperatively is very important [4].

A cohort study to possibly study the relationship between values from early postoperative functional tests compared to later in rehabilitation would have been of interest. Studies examining the diagnoses of patients with hip fracture would be of value, as different diagnoses may have different impacts on the recovery of functioning [24].

## Conclusion

The study showed that there has been a change between 2018 and 2008 in the postoperative functioning of patients with hip fracture. In 2018, walking capacity was more impaired and the number of patients with multimorbidity had increased. In 2018, the hand grip strength was better and the proportion of physically active was higher. The results may be important for reasoning about future care needs for patients with hip fracture, as the patient group is large and is feared to increase further.

## Data Availability

The datasets generated during and analyzed during the current study are not publicly available due to ethical restrictions and laws (GDPR) of disclosing personal data, authors have to seek permission to allow us to make the data used in this study available, but are available from the corresponding author on reasonable request after permission is granted from Örebro University. Inquiries for data access should first be sent to, asa.andersson@oru.se who will then contact the ethics board for permission to openly share the data.
